# Tobacco Smoke Exposure and Eustachian Tube Disorders in US Children and Adolescents

**DOI:** 10.1371/journal.pone.0163926

**Published:** 2016-10-06

**Authors:** Mira A. Patel, David J. Mener, Esther Garcia-Esquinas, Ana Navas-Acien, Yuri Agrawal, Sandra Y. Lin

**Affiliations:** 1 Department of Otolaryngology-Head & Neck Surgery, Johns Hopkins University School of Medicine, Baltimore, Maryland, United States of America; 2 Department of Environmental Health Sciences and Epidemiology, Johns Hopkins Bloomberg School of Public Health, Baltimore, Maryland, United States of America; 3 Department of Preventive Medicine and Public Health, School of Medicine at Universidad Autónoma de Madrid / IdiPaz, and CIBER of Epidemiology and Public Health (CIBERESP), Madrid, Spain; University of Missouri Columbia, UNITED STATES

## Abstract

**Objectives:**

To describe the association between active, environmental tobacco smoke (ETS) exposure and the prevalence of eustachian tube dysfunction (ETD) in the U.S. pediatric population.

**Study Design:**

Cross-sectional.

**Setting:**

U.S. representative demographic and audiometric data from the National Health and Nutrition Examination Survey (NHANES);2005–2010.

**Subjects and Methods:**

The study consisted of 2,977 children aged 12–19 years. ETD was defined as middle ear pressure <100mm H20. ETS was defined as non-active smoking in individuals with serum cotinine over the limit of detection (≥0.015 ng/mL) and <10 ng/mL(N = 1559).

**Results:**

The prevalence of ETD was 6.1%. After multivariate adjustment for age, sex, body mass index, education level, ethnicity, or having a cold, sinus problem or earache during the last 24 hours, compared to unexposed children, the odds ratios (95% confidence interval) of ETD for those exposed to ETS ages 12–15 in the first, second and third tertile of cotinine concentrations were, respectively, 1.38 (0.53–3.60), 0.99 (0.53–3.60) and 2.67 (1.12–6.34). Similarly, the odds ratios (95% confidence interval) of ETD for those exposed to ETS ages 16–19 in the first, second and third tertile of cotinine concentrations were, respectively, 1.28 (0.48–3.41), 0.99 (0.40–2.48) and 2.86 (1.19–6.88).

**Conclusion:**

These data suggest that children and adolescents exposed to high concentrations of ETS may have an increased prevalence of ETD.

## Introduction

The prevalence of cigarette smoking in the United States among adults was 17.8% as recently as 2013, with significantly high tobacco use rates (21.9%) among young men aged 18–24 [1]. High rates of tobacco use in American households over the last decade has translated to the exposure to tobacco smoke of 88 million non-smoking children and adults ≥3 years of age between 2007–2008, and of 40% of children ages 3–11 between 2011–2012, resulting in a number of adverse health effects including middle ear disease and eustachian tube dysfunction (ETD) [2–4]. It has been hypothesized that environmental tobacco smoke may lead to ETD as a result of disruption in mucociliary clearance mechanisms of the eustachian tube, chemical irritation, allergic inflammation of the mucosa in response to chemicals contained in the tobacco smoke, and overall immunosuppressive effects secondary to tobacco smoke exposure that could lead to greater susceptibility to bacterial infections of the middle ear [5]. However, a survey of the literature conducted in 1995 did not find sufficient support in prior studies for the association of passive smoking with ETD; they cited the inadequate inclusion of other risk factors, potentially biased inclusion and exclusion criteria, and inappropriate use of statistical tests as potential shortcomings of the reviewed literature [6]. Eustachian tube dysfunction often manifests as acute otitis media with effusion (OME), recurrent acute otitis media with effusion (RAOME), or chronic otitis media with effusion (COME) [7].

Early studies examining the risk factors in children for developing eustachian tube dysfunction and need for tympanostomy tube placement for the treatment of RAOME observed that children who lived in households with at least one parent who smoked were more likely to require surgical management of OME [8,9]. Several subsequent case control studies found significantly increased odds of environmental tobacco smoke exposure in children with RAOME and OME [10–14]. Large birth-cohort studies conducted in the United Kingdom and Norway corroborated findings that children with mothers who smoked had a higher risk of developing middle ear disease [15,16]. Results of recent meta-analyses also support an association between middle ear disease and environmental tobacco smoke exposure [17,18]. Indeed, it has been shown that there are elevated serum cotinine levels and impaired mucociliary clearance mechanisms in the adenoid tissue of young children with OME [19,20].

Of importance is the reconciliation of conflicting findings regarding the link between passive smoking and ETD, particularly in light of the great physical and financial costs associated with treatment of recurrent middle ear disease in children. While one study shows that a decline in clinic visits for OME from 1993–2006 may reflect a decline in environmental tobacco smoke exposure of children during those years [21], the prevalence of children exposed to tobacco smoke currently remains high [2]. Nevertheless, there have been studies that have not found an elevated prevalence of passive smoking in those with COME or surgically treated RAOME [22,23].

To resolve these conflicting findings in the literature, we utilized results of a nationally representative sample of adult and pediatric patients in the NHANES 2005–2010 cycle to examine the association between passive or active smoking and ETD.

## Materials & Methods

### Study population

NHANES is a national, population based, multi-year cross-sectional epidemiological study designed to assess the health, functional, environmental and nutritional status of the civilian, non-institutionalized United States population. We analyzed data from the 2005–2006, 2007–2008, and 2009–2010 cycles because tympanometry was measured during those cycles. This study was restricted to participants who had serum cotinine and tympanometry measurements. A total of 6,612 children and adolescents aged 12–19 years of age participated in tympanometry measurements in NHANES 2005–2006, 2007–2008, and 2009–2010 cycles. Tympanometry data was available for 91.5% of participants screened. We further excluded participants missing data, including other covariates of interest, leaving a total of 2,977 children and adolescents with complete data. The NHANES protocol was reviewed and approved by the National Center for Health Statistics institutional review board and informed written consent was provided by the study participants older than 18 years of age or guardian for participants younger than 18 years of age, which included publishing anonymous and de-identified data for public use. Written assent was provided for all participants younger than 18 years of age. Prior to National Center for Health Statistics releasing data for public use, all records and associated data were de-identified.

### Tympanometry Measurement

Tympanometry was measured using an automated tympanometer utilizing an electromagnetic impedance unit incorporating a pressure transducer to indicate changes in compliance of the tympanic membrane while simultaneously varying the air pressure in the sealed canal of the same ear. This was accomplished by measuring the sound pressure level of a probe tone introduced into the ear canal (226 Hz at 85 dB SPL), while the air pressure in the ear canal is transformed from +200 to -312 daPa. While performing this procedure, an 84-point tympanogram was recorded including middle ear pressure, compliance, and gradient. Eustachian tube dysfunction was defined as the peak mean middle ear pressure of less than -100 daPa.

### Serum Cotinine Levels

Environmental tobacco smoke exposure was assessed by measuring serum cotinine concentrations. Cotinine was measured by isotope dilution high-performance liquid chromatography atmospheric pressure chemical ionization tandem mass spectrometry (ID HPLC-APCI MS/MS) The limit of detection (LOD) for serum cotinine was 0.015 ng/ml. Serum cotinine concentrations below the LOD were replaced by the LOD divided by the square root of 2. Only participants with a history of environmental tobacco smoke (ETS) exposure were analyzed; those individuals who reported never smoking and had serum cotinine levels below the level of detection were categorized as unexposed. Those who reported never smoking and had serum cotinine levels above the level of detection, but < = 10ng/ml were divided into tertiles of serum cotinine. Young adolescents (ages 12–15) were analyzed separately from older adolescents (ages 16–19).

### Other covariates

Demographic questionnaire information included age, gender, race/ethnicity (non-hispanic White, non-Hispanic Black, Mexican American, Other), and education level of the reference person (< high school, high school or equivalent, > high school). Additional questionnaire information ascertained was whether participants had a cold, sinus problem, or earache in the last 24 hours prior to tympanometry measurement. NHANES measured height and weight during the physical examination.

### Statistical analysis

NHANES uses a complex sampling design and constructs sample weights in order to produce nationally representative data. A complex and multistage probability sampling design is used to select participants who represent the non-institutionalized civilian United States population. Survey weights are constructed for each two-year cycle, which takes into account non-response, over-sampling, post-stratification, and sampling error.

The prevalence of ETD was calculated overall and by sex, age, ethnicity, parental education, BMI percentiles and presence of sinus problems, cold or earache. The association between serum cotinine levels and ETD was evaluated using multiple logistic regression. Two models were built. The first model adjusted for age and sex, while the second was further adjusted for ethnicity, education level, weight, serum cotinine level tertile, and whether participants had a cold, sinus problem, or earache in the last 24 hours. All analyses were performed using STATA version 13.

## Results

A total of 2,977 children and adolescents aged 12–19 years of age participated in tympanometry measurements in NHANES 2005–2006, 2007–2008, and 2009–2010 cycles. Baseline characteristics are listed in Table 1. Children and adolescents with ETD were more frequently male, obese, and had a higher prevalence of sinus problems, cold or earache within 24 hours prior to tympanometry.

**Table 1:** ^a^Demographic Characteristics of children and adolescent participants by ETD, National Health and Nutritional Examination Surveys, 2005–2010 (N = 2977).

		Eustachian Tube Dysfunction	
		(+) Present (< or = -100 daPa)	(-) Absent (> -100 daPa)
		N = 182	N = 2795
**Gender**	Male,%	56.2	49.3
**Age**	Mean (SD)	14.7 (0.17)	15.1 (0.05)
**Ethnicity**	White	57.1	58.6
	Black	13.5	15.5
	Mexican	15.0	13.6
	Other	14.4	12.3
**Education**	Less than High School	21.8	18.8
	High School	25.5	21.6
	Greater than High School	52.7	59.6
**Weight**	Normal	49.7	65.7
	Overweight	17.5	16.0
	Obese	32.8	18.3
**Sinus problem, cold or earache**^b^	Present	30.1	11.6

^a^Data in table 1 are unweighted percentages for categorical variables and median (interquartile range) for continuous variables.

^b^Symptoms present or absent within 24 hours prior to tympanometry.

SD, Standard Deviation.

Median (IQR) for serum cotinine levels were 0.06 ng/mL (0.02–0.76). In total, 1,559 children and adolescents were exposed to environmental tobacco smoke based on serum cotinine levels. Using NHANES standardized population weights, 5.5% of those exposed to ETS had ETD [median (IQR) middle ear pressures -114 (-156; -36)]

Compared to unexposed adolescents, the odds ratios (95% confidence interval) of ETD for those exposed to ETS in ages 12–15 in the first, second and third tertile of cotinine concentrations were, respectively, 1.38 (0.53–3.60), 0.99 (0.42–2.30) and 2.67 (1.12–6.34); p-value for linear trend = 0.45, and in ages 16–19 were 1.28 (0.48–3.41), 0.99 (0.40–2.48), and 2.86 (1.19–6.88); p-value for the linear trend = 0.10 (Table 2).

**Table 2:** Multiple logistic regression examining the association of serum cotinine levels and eustachian tube dysfunction in children and adolescents exposed to environmental tobacco smoke from the National Health and Nutrition Examination Survey 2005–2010 Cohorts.

		Odds Ratio (95% CI)	
	N	Model 1^a^	Model 2 ^b^
**Ages 12–15**	**1696**		
**Unexposed** (≤0.015 ng/ml)	866	1.00	1.00
**1^st^ cotinine tertile:** 0.015–0.079 ng/ml	264	0.90 (0.43–1.92)	1.38 (0.53–3.60)
**2^nd^ cotinine tertile:** 0.08–0.413 ng/ml	290	0.92 (0.51–1.67)	0.99 (0.42–2.30)
**3^rd^ cotinine tertile:** 0.414–9.99 ng/ml	276	1.38 (0.70–2.70)	**2.67**^c^ **(1.12–6.34)**
P- value trend		0.46	0.45
**Ages 16–19**	**1281**		
**Unexposed** (≤0.015 ng/ml)	552	1.00	1.00
**1^st^ cotinine tertile:** 0.015–0.079 ng/ml	236	0.90 (0.43–1.92)	1.28 (0.48–3.41)
**2^nd^ cotinine tertile:** 0.08–0.413 ng/ml	263	0.92 (0.51–1.67)	0.99 (0.40–2.48)
**3^rd^ cotinine tertile:** 0.414–9.99 ng/ml	230	1.38 (0.70–2.70)	**2.86**^c^ **(1.19–6.88)**
P- value trend		0.07	0.10

^a^Model adjusted for sex and age.

^b^Model further adjusted for weight, education, ethnicity and the presence of a cold, sinus problem, or earache within 24 hours prior to tympanometry

^c^*P* < .05

## Discussion

We report that exposure to environmental tobacco smoke increases ETD as defined by tympanometry measurements in those with elevated serum cotinine levels in a nationally representative sample of children and adolescents in the United States.

Studies of prolonged tobacco smoke exposure in rats have shown a loss of cilia, squamous metaplasia, and goblet cell aplasia followed by hyperplasia in the eustachian tube mucosa over the course of months [24,25]. Smoke exposure may produce mucosal inflammation in the middle ear as evidenced by increased production of tumor necrosis factor-α in murine middle ear epithelial cells [26]. Long-term passive smoking may result in increased mucous secretion into the eustachian tube, as demonstrated in rats over a 6-month period of exposure; when combined with smoke-induced impaired mucociliary clearance of the eustachian tube, obstruction may occur resulting in middle ear disease and OME [5,20,24]. Indeed, further studies in rats have found that tobacco smoke exposure results in prolonged mucociliary clearance times and an inability to equilibrate negative middle ear pressure with increasing smoke exposure [27].

This study supports the findings of recent meta-analyses evaluating the association between middle ear disease and environmental tobacco smoke exposure, which reported an odds ratio of 1.37–1.62 of developing middle ear disease and an odds of 1.83–1.86 of undergoing surgery for middle ear disease in children who lived with a smoker [17,18]. Indeed, it has been shown that elevated serum cotinine levels are associated with a 38% higher rate of new episodes of OME in children ≤3 years of age in one study, and histopathologic analysis of adenoid tissue in passive smokers with OME has demonstrated significantly impaired mucociliary transport relative to non-smokers with OME [19,20].

Because middle ear effusion typically occurs in the setting of negative middle ear pressure caused by ETD, tympanometry is a useful tool to assess eustachian tube function [28–30]. Two separate studies of approximately 800 six-to-seven-year-old children found that passive smokers had increasingly negative middle ear pressures by tympanogram and a higher frequency of middle ear effusion in a dose-dependent manner [28,29]. The first study conducted in 1989 found a trend toward greater middle ear negative pressure by tympanometry with increasing serum cotinine concentration, with an adjusted odds ratio of 1.13 for every two fold increase in cotinine concentration [29]. The second study conducted in 1990 reported a significant trend of growing prevalence of Type B tympanograms with increasing numbers of smokers in the home (χ^2^ = 4.15, p<0.05) [28]. Our cohort demonstrated similar findings in support of the association among passive smoke exposure and increased ETD as defined by negative middle ear pressure. A primary difference between our study and the aforementioned studies is the stratification of those with ETD. We defined ETD as middle ear pressures below 100 mm H_2_O, an objective and easily reproducible measurement indicative of ETD. In contrast, prior studies have only examined the association between tympanogram type and the number of smokers in the household [28,29], which is less specific and more subjective, especially in regard to classifying tympanogram type.

Previous studies have also found a positive association between serum cotinine concentration and increasingly negative middle ear pressure [29]. Our results indicate that a serum cotinine above 0.414 ng/mL may be useful to indicate a threshold for and subsequent correlate for the presence middle ear disease for at risk children. Moreover, we demonstrate that in children exposed to ETS at high concentrations, there is a significantly higher odds of ETD. It may be that children have variable susceptibilities to the effects of environmental tobacco smoke and mucociliary clearance of the eustachian tube resulting in unpredictable degrees of ETD. Moreover, because the half-life of cotinine is up to 20 hours, children who had been exposed just prior to being tested may have higher levels of cotinine regardless of the number of smokers in the household, resulting in serum cotinine levels that are not representative of the degree of chronic tobacco smoke exposure. The metabolism of nicotine to cotinine may also vary with age, sex, diet, and inhibitors of the liver enzymes CYP2A6 responsible for nicotine metabolism [31]. The aforementioned factors may affect serum cotinine concentration and confound the relationship between ETD and cotinine levels, possibly accounting for the non-linearity of that relationship within this study.

The strengths of this study include analysis of tympanometric and smoke exposure information available in a large, nationally representative cohort of pediatric patients that allows for adjustment of a number of possible confounders. Nevertheless, inherent limitations of this study include tympanometric measurements and cotinine levels as secondary measurements of ETD and tobacco smoke exposure, respectively. Moreover, this study is cross-sectional, and therefore causality with respect to exposure variables cannot be determined. While this study attempts to estimate the prevalence of eustachian tube dysfunction in the general population, it must be recognized that the analysis is vulnerable to the limitations of selection bias, imperfect response rate, and individuals with missing data.

In all, our results suggest an association between passive smoking and ETD in children. Further investigation of longitudinal cohorts would provide greater insight into the long-term consequences of prolonged smoke exposure on eustachian tube function, especially as passive smokers age into adolescence and adulthood. Opportunities for future study include establishing a more defined relationship between environmental tobacco smoke exposure and ETD. Presently, it is unknown what cumulative dose of tobacco smoke exposure is required to produce negative middle ear pressures and subsequently ETD. Moreover, given recent insights into the possible underlying mechanism of ETD caused by smoke exposure, it may be clinically relevant to identify children that may be at a greater risk for developing ETD at lower dosages of smoke exposure, such as those predisposed to allergies and atopic disease.
